# *DAT1* 5′-Un-Translated-Region Methylation Patterns as Bio-Markers of ADHD Psycho-Pathology: Contribution to Disease Prognosis and to Monitoring of a Successful Therapy

**DOI:** 10.3390/biomedicines11092546

**Published:** 2023-09-15

**Authors:** Valentina Carpentieri, Silvia Cugno, Katarina Lockic, Esterina Pascale, Walter Adriani

**Affiliations:** 1 Center for Behavioral Science and Mental Health, Istituto Superiore di Sanità, I-00161 Rome, Italy; carpentieri.1406747@studenti.uniroma1.it; 2 Faculty of Psychology, International Telematic University Uninettuno, I-00186 Rome, Italy; s.cugno@students.uninettunouniversity.net (S.C.); k.lockic@students.uninettunouniversity.net (K.L.); 3 Department of Medical-Surgical Sciences and Biotechnologies, Sapienza University of Rome, I-00162 Rome, Italy; esterina.pascale@uniroma1.it

**Keywords:** dopamine transporter (*DAT1*) genotypes, CpGs methylation biomarker, externalizing and internalizing behavior, Attention-Deficit/Hyperactivity Disorder (ADHD)

## Abstract

Epigenetic modifications, such as changes in DNA methylation, have been linked to several diseases in recent years. The purpose of our study was to search for biomarkers that (using non-invasive techniques) could assist the clinician in the prognosis of infant/adolescent psychopathology. We previously showed that changes in methylation of the 5’-UTR in the *DAT1/SLC6A3* gene can be used as a biomarker for the prognosis of initial severe ADHD: treatment-resistant severe ADHD children were characterized by methylated CpG 1 in particular, while methylated CpGs 2 and 6 were then found in children who improved after the therapy. Further, we confirmed these outcomes and provided the hypothesis that symptomatology might be influenced by the children’s genotype and family environment. In particular, levels of CpG 3 methylation in the heterozygous ADHD children were associated with high paternal own risk or stress. Eventually, we found that the same biomarkers are more broadly useful in the field of internalizing or externalizing symptoms (when a certain vulnerability is already present in the child). In particular, it was seen how inheriting specific 9-repeat or 10-repeat VNTR alleles from the mother or from the father could modify the pattern of methylation at the 5′-UTR of the *DAT1* gene. A specific pattern of methylations (with CpG 2 following either CpGs 1 + 3 or CpG 6 at the *DAT1* 5′-UTR) has been associated, therefore, with the likelihood of an internalizing or externalizing developmental trajectory entailing ADHD-like psycho-pathological characteristics. Since each individual responds differently to a specific treatment, we suggest that these methylation patterns may be used as biomarkers to monitor the outcome and/or predict the success of a given therapy (personalized medicine).

## 1. Introduction

Epigenetic changes, particularly DNA methylation, are a promising molecular mechanism for understanding the complex interactions between genes, environment, and brain disease development [1]. The methylation status of numerous genes, in fact, is involved in the risk of developing psychopathological traits or diseases [2,3,4,5,6,7].

This commentary addresses recent advances in the use of molecular biomarkers for providing an objective account of the clinical situation in patients suffering from a very common developmental neuropsychiatric disorder, such as the attention-deficit/hyperactivity disorder (ADHD). Recent data from the group of Marzilli and Cimino as well as the group of Carpentieri and Adriani [8,9,10] suggest that a new biomarker has emerged from the dopamine transporter *SLC6A3* gene, known as *DAT1*. Taken together, the data reviewed here may be relevant to the therapeutic plan supporting children with ADHD. More specifically, we will address the potential clinical significance of correlations between methylation of the 5′-UTR in the first intron of the *DAT1* gene, on the one hand, and indicative behavioral markers of symptom severity and/or likelihood of improvement after therapy, on the other side. Herein, we will address two questions: can *DAT1* methylation markers be influenced by environmental variables such as parental behavior (paternal and/or maternal)? Can the methylation pattern be useful in predicting/determining whether a particular therapy might be successful in a particular subject?

The first study, by Marzilli and Cimino [8], addresses the correlation between methylation markers at specific CpG loci and key symptoms suggestive of ADHD; the other two works by Carpentieri and Adriani [9,10] address a purely prognostic intent, namely whether the analysis of methylation can help physicians to establish and monitor an effective treatment plan. According to these two\three papers, when merging the various details about the patient and her/his family, based on the type of methylation pattern observed, we could eventually be able to predict the initial severity and treatment resistance of ADHD and subsequently formulate individualized therapy. Conflicts within a family add vulnerability against a patient’s capacity to cope. The use of the methylation biomarker was considered in recent study by other authors (considering *DAT1* and other involved genes) as an important non-invasive method for the recognition and treatment of ADHD [11,12,13]. These works highlight the importance of personalized and precision medicine, which consists first in identifying at-risk individuals and then improving therapeutic efficacy via the administration of more specific treatments.

In addition, Carpentieri and Adriani [14] performed an experiment to investigate whether inheritance of specific 9- or 10-repeat VNTR alleles can alter the methylation pattern in CpGs islands at the 5′-UTR of the *DAT1* gene. This experiment hypothesizes that the 3’-UTR VNTR genotype may influence epigenetic modifications at the 5’-UTR of the *DAT1* gene. Four sub-groups of children were studied: *DAT1* 9/9 and 10/10 homozygous; 9/10 heterozygous, born from a 9/10 mother and a 10/10 father (the “heM” subgroup); and 9/10 heterozygous, born from a 10/10 mother and a 9/10 father (the ”heF” subgroup). For each of these subgroups, a specific methylation pattern was observed at the *DAT1* 5’-UTR. The presence of epigenetic alterations has also been associated with the presence of internalizing and\or externalizing symptoms. While the 10/10 genotype was previously associated with severe ADHD, this experiment suggests that 9/9 children exhibit internalizing symptoms (see also [15]). Interestingly, the data for the heterozygous 9/10 subjects show that what really matters is whether the father in particular has transmitted the 9R or 10R *DAT1* allele, as well as the quality of his relationship with his son/daughter. Overall, by comparing data from our three recent papers [9,10,14], this commentary proposes the use of epigenetic biomarkers for the diagnosis and/or prognosis of a prototypical externalizing disorder, such as ADHD, as well as internalizing psychopathological traits. The use of *DAT1* methylation as a biomarker to detect ADHD symptoms, as well as internalizing and\or externalizing psycho-pathological traits, is also supported by other studies [15,16,17,18].

## 2. The Importance of *DAT1* Biomarkers in the Diagnosis and Monitoring of Treatment

The main characteristics of the two studies [8,10] are summarized in Table 1. Both were conducted on Italian children aged 6 to 11/12 years. In the study by Marzilli and Cimino [8], the children’s ADHD symptoms and the affective environment provided by their mothers and fathers were examined in relation to *DAT1* 5′-UTR methylation levels. In contrast, the second study sought to identify a potential biomarker that would provide early detection of the severity of the subjects’ psychopathology. Ideally, such biomarker would allow optimization of individualized therapy by providing objective predictions on the likelihood of success for a particular treatment approach at an early stage. Thus, while the first study has a more diagnostic purpose, the other has a predictive therapeutic intent. Together, they aimed to improve diagnosis and subsequently assign an effective targeted therapy.

The two studies are similar in many ways, although, in the case of Carpentieri and Adriani [10], the ADHD diagnosis was made by a clinician (following the criteria of DSM-IV-TR), whereas in the study of Marzilli and Cimino [8], the children were recruited in the school setting and the ADHD symptoms were scored by the psychological test CBCL 6–18 DP. The study conducted by Carpentieri and Adriani [10] considers a population already diagnosed with mild or severe ADHD and wonders if *DAT1* 5′-UTR is a biomarker, potentially able to: (a) discriminate between severe and mild disease and/or (b) predict symptoms’ improvement after psychotherapeutic (cognitive behavioral therapy, CBT) or pharmacological (methylphenidate, MPH) therapy. In the study by Marzilli and Cimino [8], the aim was to examine children’s emotional-behavioral functioning and ADHD-like symptoms, by considering parents’ psychological profiles, parental stress, and marital adjustment. The *DAT1* gene (responsible for transporting dopamine at the neuronal level, a mechanism known as reuptake) was considered. Two regions were examined on this gene: the 5’-UTR in the first intron and the 3’-UTR VNTR sequence, located downstream of the coding sequence of the gene. The latter is a polymorphic region that allows different alleles to be distinguished: the most common are those with 10 repeats (10R, therefore referred to as 10/10 in the case of homozygosity) or 9 repeats (9R; when at least one such allele is carried, independently of the other, they are referred to as 9/x).

In the study conducted by Carpentieri and Adriani [10], a population of 60 children (diagnosed with ADHD) was examined for possible methylation and de-methylation of individual CpG loci at the 5′-UTR of the gene. Positive correlations of each CpG with all possible CpG pairs were found between subjects with severe and treatment-resistant conditions, when compared to improving subjects after the administration of therapy. This could likely be due to epigenetic and/or environmental changes. Individuals with a severe form of ADHD were characterized by a “seesaw” trend within a methylation triplet, i.e., a correlation pattern in which certain CpGs switch from a single correlation to a multiplicative correlation [10]. When patients improve after the administration of therapy, we never find this type of “seesaw” but only CpGs that are always correlated in a somewhat stable fashion, either as single or as multiplied pairs. 

To sum up, the take-away message is clear. Methylation in CpG 2 and\or CpG 6 could be a discriminatory biomarker in identifying an improving subject, while methylation of CpG 1 could serve as a biomarker to identify subjects with severe and treatment-resistant ADHD [9].

The study by Marzilli and Cimino [8], on the other hand, sought to determine whether there was a relationship between the genetic influences of ADHD and environmental influences, such as the quality of the family relationship (particularly, the relationship between father and mother, their perceived stress towards the child, and their own risk for psychopathology). The sample consisted of subjects aged 6 to 11 years (who had not yet been diagnosed with ADHD) to determine whether there might be an association with characteristics suggestive of an ADHD-like disorder (self-report of possible susceptibility assessed with the DSM-5 oriented ADHD Problem Scale and CBCL DP). An initial analysis using buccal swabs divided the subjects into two main *DAT1* subgroups: 9/x genotype and 10/10 genotype. Then, methylation of specific CpG sites was analyzed to search for associations with ADHD syndrome. Specifically, CpG 1 methylation was positively associated with ADHD symptoms in subjects with 10/10 genotype, whereas in contrast, CpG 6 methylation was associated with low ADHD levels in subjects with 9/x genotype.

Note that CpG 1 is associated with CpG 3, while CpG 2 makes the difference. It contributes to disease severity when associated with CpGs 1 and 3, whereas it predicts recovery when associated with CpG 6 [14]. Marzilli and Cimino [8] wondered whether other environmental factors, such as maternal and paternal influence, might play a significant role in methylation of *DAT1* 5′-UTR loci, and consequently influence ADHD symptoms.

The results of the study by Marzilli and Cimino [8] can be summarized as follows:

- CpG 1 site methylation was positively associated with maternal psychopathological risk and negatively associated with paternal dyadic adaptation, two somewhat related parameters. This was true only for 10/10 genotype, whereas there was a non-significant association for 9/x genotype.
- Note that for CpG 2 site methylation, at least for 9/x children, it makes a difference whether it was associated with CpGs 1 and 3 or with CpG 6, the latter affecting recovery. As for the paternal role, low levels of this site’s methylation in 9/x children were associated with high paternal stress, indicating that a stressed father may impede recovery. On the other hand, there was a positive correlation with maternal perception of dyadic adaptation for 9/x children (but not in 10/10 children), confirming that maternal discomfort with paternal stress contribute to the maintenance of low CpG 2 methylation!
- In 10/10 children, methylation of the CpG 3 site was predicted by maternal stress (i.e., not maternal risk in general, but perceived problems in the relationship with the children), with a non-significant association for the 9/x subgroup: as a matter of fact, vulnerability of 10/10 genotype is dependent on CpGs 1 and 3 methylation via maternal stress added on top of own risk. Interestingly, methylation of the CpG 3 site was predicted by father’s high own psycho-pathological risk (GSI score) in 9/x children but not in 10/10 ones.
- Finally, high levels of CpG 6 site methylation in 9/x genotype were associated with high paternal P-CDI score (indicating poor father–child relationship), but not in 10/10 genotype. To sum up the latter data, 9/x children of stressed fathers did not recover after therapy, because of an opposed fate between CpGs 2 (and 3) when compared with CpG 6.
- Greatest vulnerability is present when the CpG 3 methylation is high because mothers are heavily unhappy with their 10/10 children. For 9/x children, the father’s own risk or his child-related stress play a key role. *A series of considerations can therefore be put forward.* We hypothesized the following picture: that high paternal infant-related stress on one side, or maternal own risk on the other hand, acting on 9/x or 10/10 genotype respectively, might increase methylation at CpGs in the dynamic interaction of CpG 3 or 2 with CpG 1 or 6.
- In the father–child dysregulation profile, the *DAT1* 9/x genotype actually moderated the relationship: a low CpG 3 methylation (in the child) was associated with increased paternal stress, whereas a significant positive association was found between high CpG 3 methylation and paternal GSI, a sign of own risk in the father himself. Accordingly, these associations with the 9/x children could be moderated by the paternal phenotype.

Therefore, we can formulate a hypothesis: Dynamic methylation and de-methylation of 5′-UTR loci may occur in the same or opposite manner depending on the paternal relationship with the child. In other words, the opposite fate of CpGs 2 and 3 (anti-correlation; [10,14], the former following CpG 6) can be observed when the at-risk father has a good dynamic with the child, with a recovering ADHD observed in the latter. On the other hand, the same fate can be observed at CpG 2 and 3 (correlation; [10,14], both following CpG 1) when the stressed father has real discomfort with the child, so that ADHD remains severe. DA levels in the mesolimbic pathway, which in turn may predispose children to impulsive behavior, may be fine-tuned by *DAT1* expression, which in turn could be modulated by altering CpG 2 vs. 3 dynamics [19]. Fathers could thus cause emotional and behavioral dysregulations in children via an influence on the 5′-UTR of *DAT1*.

## 3. The Importance of the 3′UTR (VNTR) in the Dynamic Methylation of The *DAT1* Gene

The evidence described above provides us with new insight into how the most common alleles of the human *DAT1* genotype (the 40 bp variable number of tandem repeats, VNTR, in the 3′ UTR of the *DAT1* gene) affect methylation dynamics at the 5′ UTR of the *DAT1* gene, an epigenetic modification associated with ADHD [10,14]. The 3′-UTR VNTR alleles with 9- and 10-repeats were apparently moderated by the father and the mother, respectively. This idea prompted a new study aimed at testing whether the dynamics of methylation at the 5′-UTR of the *DAT1* gene would be altered by the presence of either allele [10,14].

Given the contribution of fathers to heterozygous children, it is obviously critical to know whether they had either a maternal (heM), or paternal (heF), 9-repeat allele. In total, 122 children with an average age of 6 years were recruited, from which subgroups were formed. In the first run, 9/9 and 10/10 homozygous children were selected and compared. In the second run, only heterozygous children, in whom it was possible to distinguish the allele inherited from the mother or the father, were analyzed. This procedure is only possible when analyzing heterozygous individuals descended from one homozygous and one heterozygous parent. For this purpose, two subgroups were formed [14]: The heM subgroup consists of 9/10 children born from 9/10 heterozygous mothers and 10/10 homozygous fathers; the heF subgroup consists of 9/10 children born from 10/10 homozygous mothers and 9/10 heterozygous fathers. According to Mendel’s laws, heM children inherit allele 9R from their mothers and 10R from their fathers; the reverse is true for heF children. 

In 10/10 homozygous children, the methylation in CpG 6 correlates negatively with all combinations of the other CpGs. When CpG 6 is methylated, the other CpGs are de-methylated, and vice versa. Furthermore, in the dynamics of methylation found in 10/10 homozygous children, there is a ‘seesaw’ phenomenon that has been described previously and that is also found in ADHD children. This particular seesaw affects CpGs 3 and 5. In the first result, CpG 3 is correlated as a single, and CpG 5 is within the multiplied pair. In the following result, the two CpGs are reversed: CpG5 is now correlated as a single, while CpG3 is multiplied within the pair. To complete the correlation triplets, methylated CpGs 1 and 2 were always within the multiplied pair [14]. In summary, in 10/10 children, the block of CpGs 1, 2, and 3 behave as a whole. This goes along with the mother’s own risk plus stress for her child with ADHD [8].

In 9/9 homozygous children, the seesaw is found again between CpGs 3 and 5. Here we can observe de-methylated CpGs 1 and 6 and methylated CpG 2, completing the correlation triplet. Thus, the major difference between 9/9 homozygous children and 10/10 ones is between CpGs 1 and 6, which are clearly opposite. In summary, the CpG 1 + 2 + 3 block is absent in 9\9 kids, and there were virtually no ADHD children with a 9/9 genotype in our data set. The 9/9 homozygous children are the unique subgroup characterized by internalizing symptoms [14].

The heM subgroup is characterized by a new seesaw between CpG 1 and CpG 3, both of which are methylated. To complete the triplet, in this case, we have one of CpGs 2, 5, and 6, which is always de-methylated. Here, the fates of CpGs 1 and 6 are opposite, as was also observed in 10/10 homozygous children, but now CpG 2 apparently follows the fate of CpG 6 rather than CpG 1. Therefore, we suspect that this pairing of the fates of CpG 2 + 6 is a biomarker for recovery [9]. The maternal 9R allele might be the protective factor, as 9/9 children do not have ADHD in our hands. 

In the heF subgroup, there is another seesaw between CpGs 3 and 6, where both transition from de-methylated, when correlated individually, to methylated, when correlated as a pair. When the triplet was completed, de-methylated CpGs 1 and 5 and methylated CpG 2 were found [14]. Here, the pairing of fates between CpGs 2 and 6 is unstable and transient. Since the maternal allele is the 10R allele, the father plays a crucial role not only in the transmission of the 9R allele but also in whether or not he contributes to ADHD by showing child-related stress or not!

Therefore, heterozygous ADHD patients may benefit from therapies and show rapid recovery if their mother, but not the father, carries and transmits the 9-repeat allele. Instead, increased methylation at CpG 1 was found in all the ADHD children with initial and/or treatment-resistant severe pathology. This was found in the 10/10 children and in the 9/x children when determined by a vulnerable methylation setup (namely, when CpG 1 rises and CpGs 2 + 6 fall). Thus, treatment-resistant severe ADHD may result from the inheritance of two 10-repeat alleles or (in heF) from the influence of the mothers’ 10R allele (9/9 and heMs are protected). If a child inherits a DAT 9R allele from the father, it can make a difference whether or not the father enters in synthony with his child: fathers can help by tuning their behavior with that of their children.

## 4. Missing Endpoint for an Ideal Experimental Design

Methylation is one of the most important among epigenetic modifications regulating gene expression. We are well aware of the limitations of our own work because methylation levels are not only related to a particular psychopathological trait but also to the amount of mRNA produced. To investigate the possible interference between methylation in a specific gene region and psychopathological traits, an ideal experimental design was published by Rizavi and colleagues [20]. The human GR gene (*NR3C1*) has a very complex gene structure with multiple 5′ untranslated regions (5′-UTRs), consisting of nine non-coding exons, seven of which are characterized by CpG islands. A sample made of 24 brains from healthy, non-psychiatric control subjects and 24 suicide bombers was analyzed postmortem. Like us, Rizavi and colleagues [20] examined changes in methylation levels at the 5′-UTR of *NR3C1*. In addition, they determined the RNA expression profiles of all variants expressed in this region, as well as the expression levels of DNA methylating (DNMT1, 3A, and 3B) and de-methylating enzymes (TET1, 2, 3 and GADD45α, β, γ). The authors provide evidence that methylation changes can regulate the expression of GR and that this contributes to teen suicide.

In contrast, our studies do not analyze mRNA expression and protein activity levels of (de)methylation enzymes, which unfortunately makes it quite difficult to draw more definitive conclusions. However, there are several other limiting factors that must be considered in both the studies here compared [8,10]:

- The sample size is small and refers to a limited geographical area, which in both cases is central Italy. We do not know whether the study would yield the same results in other countries and/or with other ethnic groups. Moreover, a larger sample would be needed to confirm the results.
- The sample was collected only once, and data do not provide meaningful information about development over time: a repeated sampling would allow the observation of potential changes over time in the dynamics of all *DAT1* CpGs. The developmental trajectory from childhood to adolescence should also be assessed, taking into account contamination by other psychiatric co-morbidities. Therefore, to consolidate the obtained results, longitudinal experiments must be conducted.

## 5. Conclusions

As a result of both the studies here compared [8,10], we can conclude that the *DAT1* gene may play an important role in identifying markers in ADHD: It can be used as a predictor of more or less severity of the disorder (in terms of rigidity vs. flexibility of behavioral symptoms), depending on the particular dynamic methylations it undergoes at its 5′-UTR. It is particularly sensitive to external environmental influences, such as a dysfunctional paternal input, to which a genetically susceptible child may well be exposed. External parental influences act as epigenetic moderators that can alter gene methylation, expression, and subsequent disease progression.

Our study proves that common features between *DAT1* 5′-UTR CpG loci can be found in members of ADHD subgroups, namely severe and treatment-resistant compared with improving patients, independent of genotype and familial environmental influence. It has been shown that molecular methylation analysis during therapeutic follow-up can provide useful feedback on whether or not the ADHD patient is actually improving. However, it has not yet been possible to use objective molecular data to confirm or dismiss our hypothesis about paternal influence, especially in the case of childhood stress expressed by fathers toward heterozygous offspring.

These studies have given importance to the role that *DAT1* plays in a specific psycho-pathological condition, such as ADHD in children. The role of *DAT1* 5′-UTR methylations in determining the severity of the disorder, and its potential improvement after therapy, is of undeniable importance. Dynamic changes in methylation profiles are a new line of research that could lead to discoveries of considerable importance in the clinical diagnostic field. It is a realistic biomarker for intervention that (if further explored) could yield significant benefits in terms of treatment timing and efficacy.

## Figures and Tables

**Table 1:** Comparison between the two studies carried out during years 2021–2022.

Study	Marzilli and Cimino, 2023 [8]	Carpentieri and Adriani, 2023 [10]
**Purpose**	*DAT1* genotype and methylation: is there a link between ADHD symptoms in children and the affective environment of mothers and fathers?	Methylation dynamics on the 5′-UTR of *DAT1* gene: can it be used as a biomarker to detect treatment success in children diagnosed with ADHD?
**Setting**	Public elementary schools in Central Italy	Child Neuro-Psychiatry Unit, University of Tor Vergata, Rome, Italy
**Subjects**	76 children aged 6 to 11 with biological parents	60 children aged 6 to 12 years old
**Criteria of Inclusion**	Families could have more than one child but had to choose only one for research	Diagnosis of ADHD according to the criteria of the DSM-IV-TR
**Independent Variables**	Maternal affective environment (P-CDI *mother*), paternal affective environment (P-CDI *father*), and the relationship between parents (DAS)	Methylphenidate (MPH), or cognitive behavioral therapy (CBT), for at least 6 weeks after reaching effect
**Relevant Results**	Children’s psychopathological risk is associated with both *DAT1* 5′-UTR methylation and paternal variables. Specifically, high ADHD scores were associated with high levels of methylation at the CpG 1 site and low levels of methylation at the CpGs 2 and 6 sites (in the children).	Dynamic methylation of specific CpG sites predicts potential improvement of ADHD after MPH or CBT therapy. Specifically, methylation at CpG 1 was found in children with severe ADHD who did not improve after therapy, whereas children with methylation at CpG 2 and 6 sites showed successful recovery of ADHD symptoms.

## Data Availability

No new data were created. However, data are available according to the respective Data Availability Statements (please refer to the original papers for more details).
